# Severe Acute Porphyria Exacerbation Post Intravenous Iron Infusion: A Case Report

**DOI:** 10.7759/cureus.68829

**Published:** 2024-09-06

**Authors:** Tarek Hammad, Sayeed Hossain, Tanweer Ahmad

**Affiliations:** 1 Department of Cardiology, Northampton General Hospital NHS Trust, Northampton, GBR; 2 Department of Internal Medicine, East Suffolk & North Essex Foundation Trust, Suffolk, GBR; 3 Department of Internal Medicine, Northampton General Hospital NHS Trust, Northampton, GBR

**Keywords:** vesicular rash, duodenal ulcers, ferrochelatase deficiency, photosensitivity, porphyrin, intravenous iron infusion, erythropoietic porphyria

## Abstract

This case report describes a 28-year-old man with erythropoietic porphyria (EPP). After receiving an intravenous iron infusion, he experienced a significant acute aggravation of his condition. The patient had a vesicular rash on the face and arms with severe itching and burning feelings in addition to nausea, vomiting, and black-colored vomit. Abnormal liver function tests and anemia were found in the lab tests. Quick diagnosis and multidisciplinary care from dermatology, gastrointestinal, and hematology experts were essential. Strict light avoidance, symptom management techniques, and cessation of intravenous iron were all part of the treatment plan. The patient's symptoms subsided over a period of 12 months, and he resumed his regular activities. In managing EPP, key learning points stress the importance of vigilance in spotting trigger variables, prompt diagnosis, light avoidance, consistent follow-up, and genetic counseling.

## Introduction

An uncommon autosomal recessive condition called erythropoietic porphyria (EPP) is characterized by a ferrochelatase deficiency that causes porphyrins to build up and cause phototoxic skin responses [1,2]. Acute porphyria flare-ups can be prompted by trigger factors, such as drugs or therapies that boost heme production [3]. Strict light avoidance, symptom alleviation, and multidisciplinary treatment are all part of management. The severity of acute exacerbations in EPP patients can be reduced with awareness of trigger events and prompt treatments. The difficulties in recognizing and treating acute porphyria flare-ups are highlighted in this case, underscoring the value of preventive interventions, routine follow-up, and genetic counselling in enhancing patient outcomes.

## Case presentation

Presenting complaints

**Figure 1:**
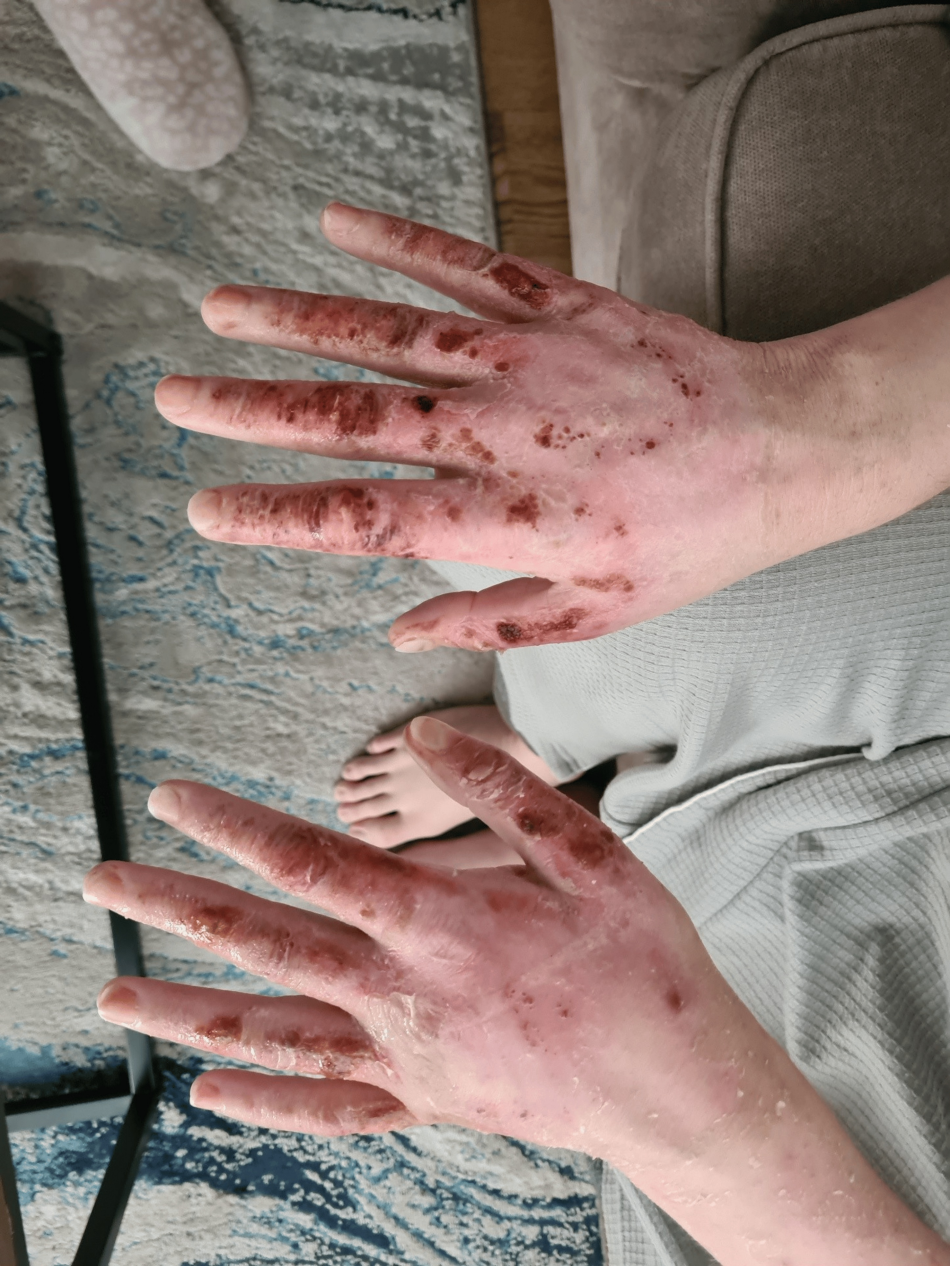
Cutaneous erythematous and erosions post ruptured vesicles in erythropoietic porphyria flare

**Figure 2:**
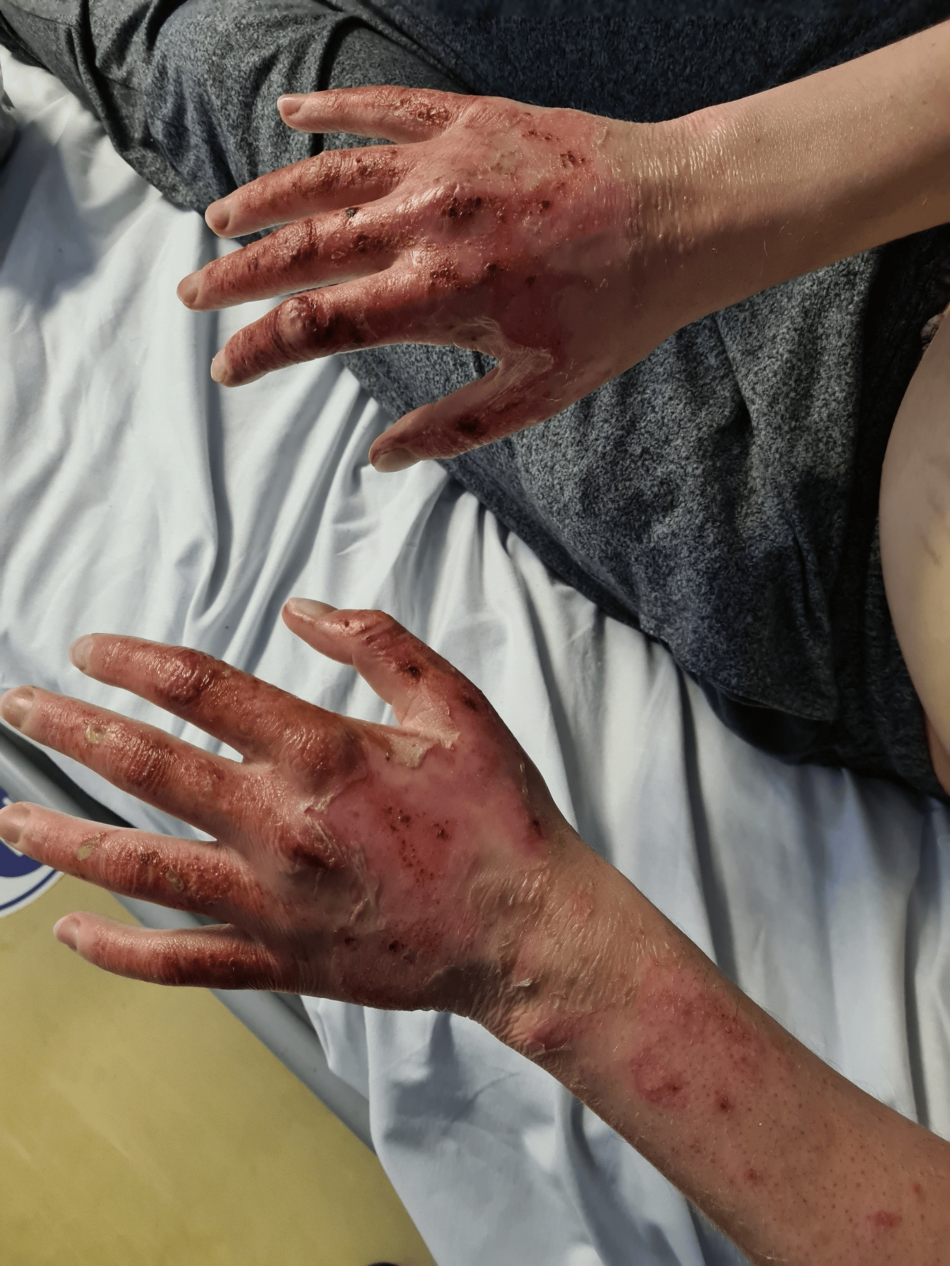
Cutaneous erosions and desquamation post ruptured bullous in erythropoietic porphyria flare

**Figure 3:**
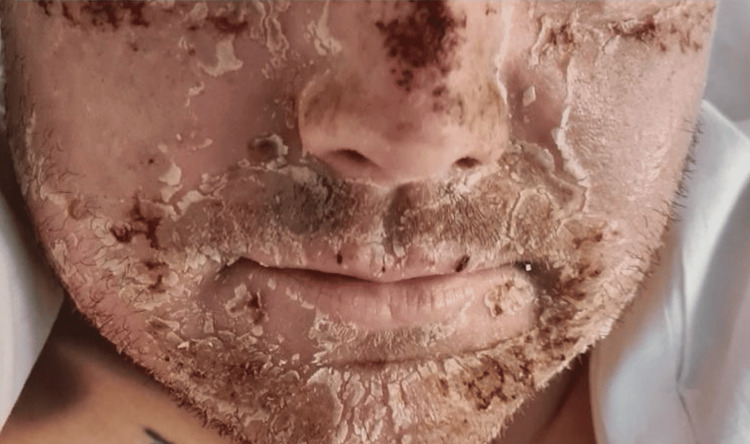
Cutaneous manifestation of erythropoietic protoporphyria flare on the face

A 28-year-old White British male arrived at our local district hospital in the emergency department complaining of feeling ill, nausea, and vomiting. He claimed to have occasional episodes of black tarry stools and vomit. Two days prior, he had an iron transfusion (ferric carboxymaltose) for chronic anaemia. He had a history of EPP, depression, and hay fever but no recent episodes of porphyria-related flare-ups since infancy. The patient denied fever, flu-like symptoms, diarrhoea, or urinary issues, and there was no recent travel history. He had no prior history of drinking or illicit drugs. The patient initially complained of feeling ill, nausea, and several episodes of vomiting, some of which included vomit that was a dark colour. He described the recent passage of black tarry stools even though he denied having fresh blood in his vomit or stool. Physical examination revealed that the patient was pale and jaundiced, with yellowish hands, face, and sclera. Upon abdominal examination, the right upper quadrant was painful, and there was an element of hepatomegaly (about three fingerbreadths below the costal edge). Examinations of the cardiovascular and respiratory systems were unremarkable. The patient's face and arms both displayed erythematous changes along with scattered vesicular rash, which ruptured and turned to erosions over the next few days (Figures 1-3). He described the prickling, scorching, and burning-itching sensation as "fire on the skin." He routinely applied water and moisturiser to treat these problems.

Relevant history

The patient had been diagnosed with EPP since childhood. He took iron supplements for his chronic anaemia but later discontinued them as it was poorly tolerated. Therefore, it was switched to intravenous infusions. On further questioning regarding the history, the patient mentioned that he had the intravenous iron transfusion one day before developing the current symptoms.

Investigations

**Figure 4:**
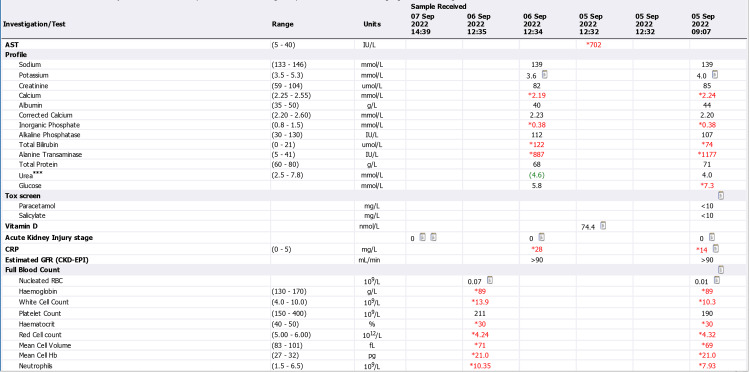
Admission and discharge lab reports (September 5 to September 7) CRP: C-reactive protein; GFR: glomerular filtration rate; CKD: chronic kidney disease; EPI: exocrine pancreatic insufficiency; RBC: red blood cells

**Figure 5:**
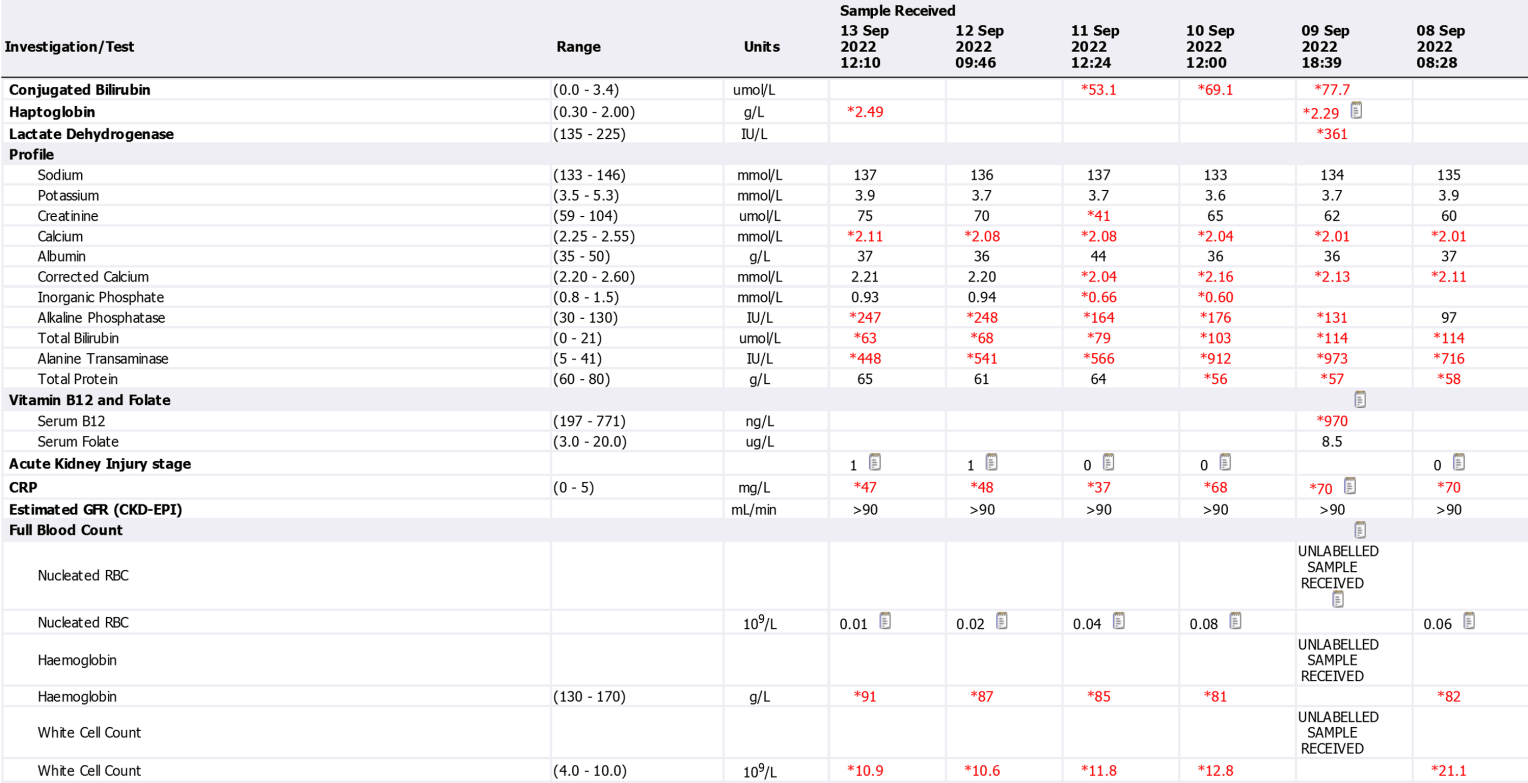
Admission and discharge lab reports (September 8 to September 13) CRP: C-reactive protein; GFR: glomerular filtration rate; CKD: chronic kidney disease; EPI: exocrine pancreatic insufficiency; RBC: red blood cells

**Figure 6:**
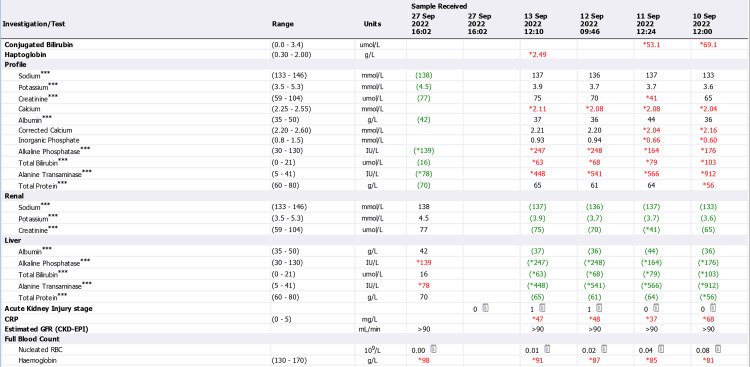
Admission and discharge lab reports (September 13 to September 27) GFR: glomerular filtration rate; CKD: chronic kidney disease; EPI: exocrine pancreatic insufficiency; RBC: red blood cells

Acutely high abnormal liver enzymes were noticed in laboratory tests, along with an elevated conjugate with bilirubin at 77.7 μmol/L (normal range: 0-3.4 μmol/L), alanine transaminase (ALT) at 1177 U/L (normal range: 5-41 U/L), and aspartate aminotransferase (AST) at 702 U/L (normal range: 5-40 U/L). The patient also displayed persistent microcytic hypochromic anaemia, which was consistent with iron deficiency anaemia (IDA) (Figures 4-6).

**Figure 7:**
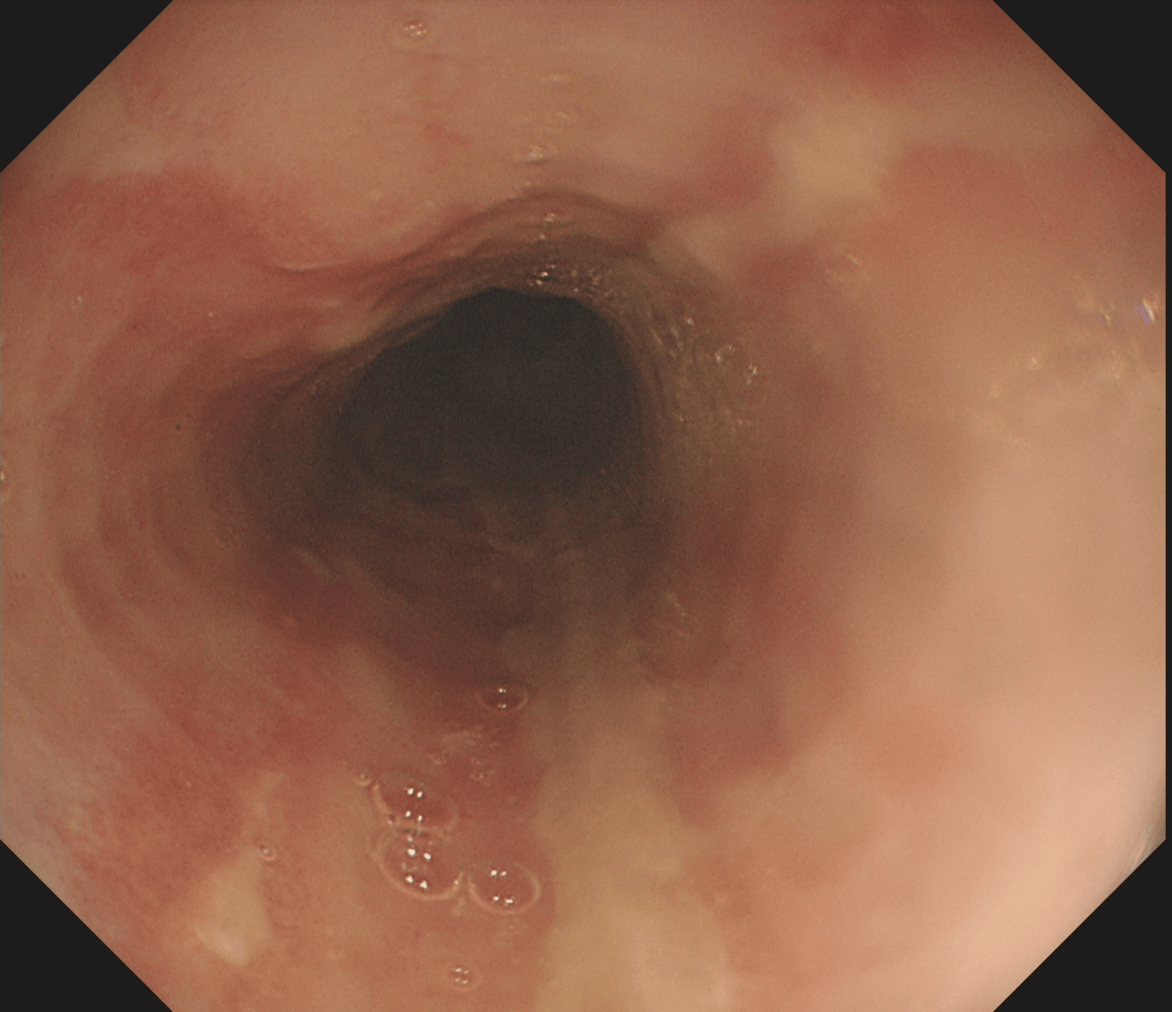
Extensive oesophagitis in erythropoietic porphyria flare seen by gastroscopy

**Figure 8:**
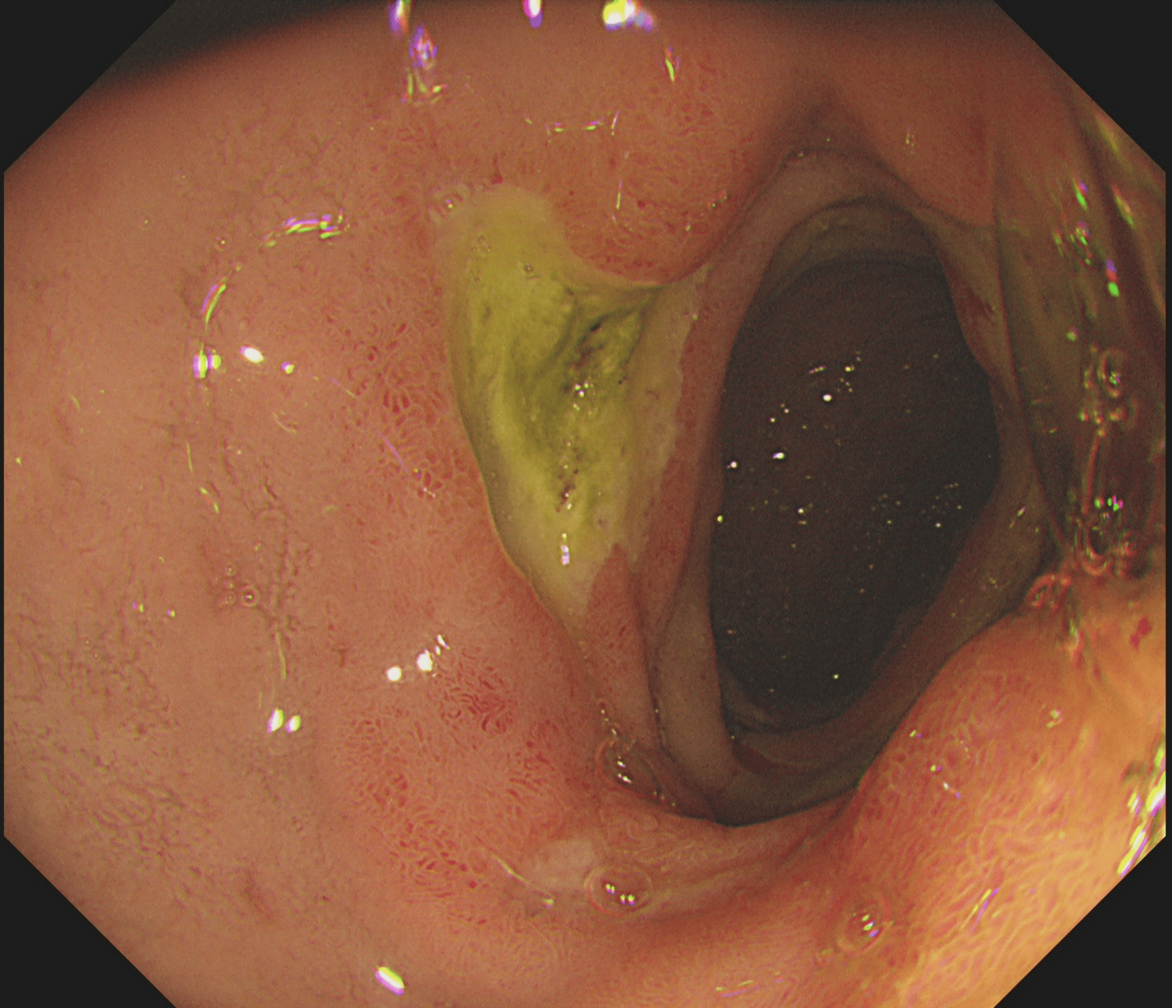
Duodenal ulcers in erythropoietic porphyria flare seen by gastroscopy

**Figure 9:**
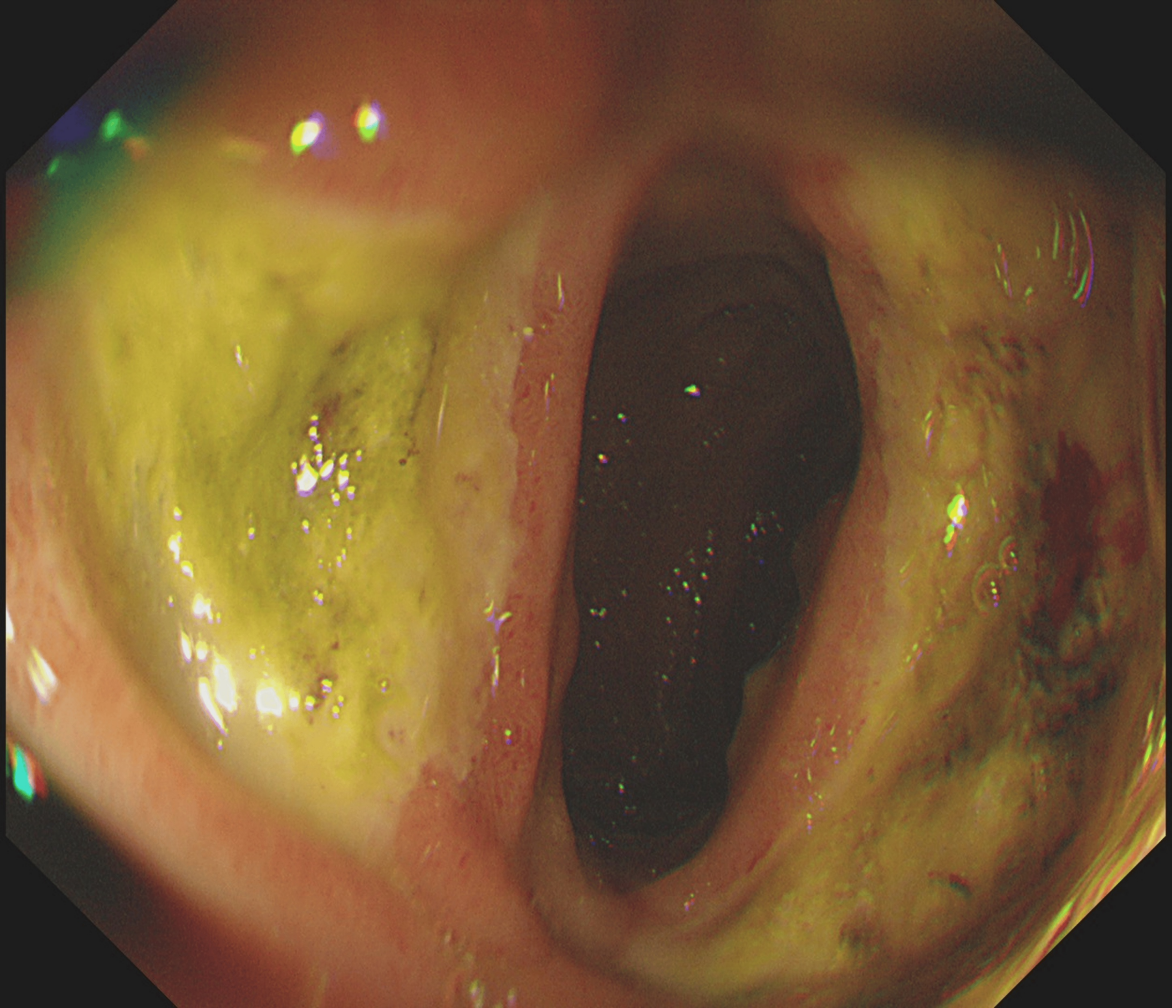
Duodenal ulcers in erythropoietic porphyria flare seen by gastroscopy

Markers of infection were only marginally raised. Hepatitis screening, autoimmune uscreening, and sepsis screening were negative. Paracetamol and salicylate levels were within normal ranges. Multiple gallstones were found in the gallbladder by abdominal ultrasonography. In light of the abnormal liver enzymes and the anaemia, a gastroscopy was performed to check for gastrointestinal bleeding, and it showed extensive erosive oesophagitis and multiple duodenal ulcers (Figures 7-9). Proton pump inhibitors (PPI) at high doses were started for the patient (pantoprazole IV 40 mg twice a day (BD)), and an endoscopic follow-up appointment was scheduled six weeks later. The dermatology department advised conservative management by removing the triggering factor and using periodic cold sponging, ice packs, and moisturiser in addition to being kept in a dark environment. Additionally, sun protector factor (SPF) >30 sunscreen and topical corticosteroids (moderately potent corticosteroid clobetasone butyrate 0.05%) were also prescribed. The patient's symptoms started to get better gradually over the next few days. He no longer needed cold sponging, moisturising, or painkillers. The itching and burning feelings disappeared, and the rash on his face and arms dramatically improved. His liver function tests gradually returned to normal levels (Figure 6). He was strongly advised against using intravenous iron infusions in the future and to keep up his sun safety routine. The patient was discharged after nearly two weeks.

Diagnosis and follow-up

A final diagnosis of acute flare of EPP with accompanying hepatopathy precipitated by intravenous iron infusion was reached based on the patient's medical history, clinical manifestations, and studies. Red cell protoporphyrin levels were found to be highly elevated during a porphyria screening, which was consistent with EPP hepatopathy. The patient has been monitored for a full year from their original presentation. During that period, the patient's health status significantly improved. The patient was followed up after discharge in a tertiary centre for metabolic diseases.

## Discussion

Extreme photosensitivity following brief and restricted sun exposure is the main symptom of EPP sufferers. Protoporphyrin, which has accumulated in their red blood cells and been deposited in cutaneous blood vessels within an hour of sun exposure, is photoactivated and causes acute skin burning, erythema, and itching. Skin abnormalities are typically detected in sun-exposed areas, including the nose, cheeks, and dorsum of the hands. The amount of sun exposure is related to how severe the dermatological effects are [4,5]. Patients with EPP are more likely to be vitamin D deficient and have a higher risk of getting osteoporosis since they tend to avoid sunlight.

For EPP patients, extracutaneous sequela, such as liver injury, is an uncommon but serious consequence. Protoporphyrin accumulation in the liver, which has cholestatic and hepatotoxic effects, maybe the aetiology of this illness. In turn, this encourages protoporphyrin accumulation in patients with quickly progressing liver conditions like severe chronic cirrhosis or catastrophic hepatic failure. Pigmented gallstones are an EPP adverse effect that is more frequent. Bile may cause the lipid-soluble protoporphyrin to crystallise. These patients are more likely to develop gallstones early in life because of cholestatic abnormalities in the liver [6]. 

Pathophysiology and mechanisms

The case studied here demonstrates the complexity of EPP and the possibility of abrupt exacerbations brought on by things like iron infusions [7, 8]. A lack of ferrochelatase, the last enzyme in the heme biosynthesis pathway, causes the uncommon autosomal recessive condition EPP to flare. Porphyrin intermediates, in particular protoporphyrin IX, accumulate as a result of this deficit in a variety of tissues, including the skin. These stored porphyrins become active when exposed to light, especially visible light, which causes phototoxic responses and the recognisable skin signs found in EPP patients.

In this instance, an intravenous iron infusion caused the patient's acute flare-up of EPP. Understanding the process causing this aggravation is crucial. Due to an underlying ferrochelatase shortage, the injection of iron most likely boosted heme synthesis, which in turn caused an accumulation of porphyrins. As a result of this excess porphyrin reaction with light, the patient's skin developed a severe vesicular rash, burning sensations, and erythematous changes. The acute hepatopathy seen could also have been caused by a hepatotoxic impact of the porphyrins [9-11].

Some pharmacological treatments were suggested, such as beta-carotene, as it might reduce photosensitivity in EPP, but a controlled trial did not provide strong evidence of its effectiveness. Other antioxidants, such as vitamin E, do not offer benefits to protoporphyria patients, and beta-carotene is unlikely to alleviate photosensitivity in other types of porphyria [12].

More importantly, porphyrinogenic agents must be strictly avoided, as they have the same aggravation process. Classical examples are antiepileptic drugs phenobarbital, phenytoin, and carbamazepine antibiotics such as chloramphenicol, rifampicin, pyrazinamide, ethambutol, and erythromycin [13].

Clinical recommendations/take-home messages

Following established clinical standards is necessary for the management of EPP and acute porphyria flare-ups. Important suggestions are presented below.

Vigilance in Identifying Trigger Factors

Acute exacerbations in patients with EPP may be caused by a variety of events; thus, healthcare professionals should be vigilant in recognising potential triggers. In this instance, a significant flare-up was brought on by an intravenous iron injection. When prescribing drugs or other therapies for EPP patients that could enhance heme production, clinicians should take the possibility of aggravation into account.

Management

Management should be conducted by a multidisciplinary team (MDT) that includes a gastroenterologist, dermatologist, and haematologist. The patient should be transferred to a specialist centre after stabilising the acute phase. Symptomatic management includes the use of topical corticosteroids, moisturisers, and cold sponging to treat the symptoms of cutaneous manifestations, such as itching and discomfort.

Avoiding Triggers

It's important to recognise and stay away from triggers, such as specific drugs (porphyrinogenic) as discussed above and iron infusions, in order to prevent acute exacerbations. Advising patients to avoid fasting or severely hypocaloric diets and alcohol intake should be nil or modest and to avoid psychological stress or exhaustion [12].

Regular Follow-up

To evaluate porphyria status and liver function, patients should have regular follow-up care from dermatologists, haematologists, and gastroenterologists.

Genetic Counselling

To determine the likelihood of passing on the EPP gene, affected individuals and their families should be provided with genetic counselling.

## Conclusions

This case study highlights the importance of a multidisciplinary approach in managing EPP and preventing acute flare-ups. Severe reaction following an intravenous iron infusion underscores the necessity of identifying and avoiding potential triggers in EPP patients. Effective management strategies, including strict light avoidance, symptom relief, and regular follow-up, are crucial in reducing the effects of this condition. The significant improvement in the patient's health over a year demonstrates the effectiveness of comprehensive care and the need for continuous monitoring. This case reinforces the value of prompt diagnosis, appropriate intervention, and preventive measures in enhancing patient outcomes and quality of life in EPP.
